# Exploring the Evolving Role of Scopolamine in Pharmacotherapy: From Cognitive Impairment to Neuroplasticity?―A Narrative Review

**DOI:** 10.3390/molecules31071219

**Published:** 2026-04-07

**Authors:** Jakub Kukla, Piotr Olejnik, Kaja Kasarełło

**Affiliations:** 1 Laboratory of Centre for Preclinical Research, Chair and Department of Experimental and Clinical Physiology, Medical University of Warsaw, 02-097 Warsaw, Poland; jakub.kukla@student.wum.edu.pl (J.K.); piotr.olejnik@wum.edu.pl (P.O.); 2 Department of Neurology, Medical University of Warsaw, 02-097 Warsaw, Poland

**Keywords:** scopolamine, muscarinic acetylcholine receptors, cognitive impairment, antidepressant effects, neuroplasticity

## Abstract

Scopolamine, also known as hyoscine, is a naturally occurring tropane alkaloid derived from plants of the Solanaceae family. Clinically, the compound has long been used for the prevention of motion sickness and postoperative nausea and vomiting, as well as for ophthalmological procedures requiring mydriasis and cycloplegia. However, beyond these established indications, increasing attention has been directed toward its broader neuropharmacological actions. This narrative review aims to summarise current knowledge regarding the pharmacological properties of scopolamine, with particular emphasis on its mechanisms of action and emerging implications in neuroscience and neuropsychiatric disorders. Scopolamine acts as a non-selective antagonist of muscarinic receptor subtypes M1–M5, interfering with cholinergic neurotransmission. Experimental and clinical studies demonstrate that scopolamine induces transient cognitive impairment. This property has led to its widespread use as a pharmacological model of Alzheimer’s disease, enabling investigation of cholinergic contributions to cognitive decline. More recently, several early clinical studies suggested that intravenous administration may produce rapid reductions in depressive symptoms, possibly through modulation of glutamatergic neurotransmission and activation of mTORC1-dependent synaptic plasticity pathways in the prefrontal cortex. Nevertheless, subsequent trials have yielded inconsistent results, and the therapeutic relevance of these findings remains uncertain. Current evidence indicates that scopolamine’s neuropsychiatric effects likely arise from complex interactions between cholinergic, glutamatergic, and neurotrophic signalling systems. Taken together, scopolamine represents both a valuable experimental tool for studying cholinergic function and a mechanistic framework for the development of novel therapeutics targeting rapid neuroplastic processes in neuropsychiatric disorders.

## 1. Introduction

Scopolamine, also known as hyoscine, is a tropane alkaloid naturally found in various genera of the Solanaceae family, particularly within the subfamily Solanoideae, including Solandra, Latua, and Mandragora genera, as well as in the Nicotianoideae subfamily [1]. It is classified as a secondary plant metabolite, primarily extracted from henbane plant (*Hyoscyamus niger*), though it can also be sourced from corkwood tree (*Duboisia* spp.) [2,3,4]. Due to its anticholinergic properties exerted on both the peripheral and central nervous systems (CNS), it is widely utilised across multiple therapeutic indications [1,4]. Pharmacodynamically, scopolamine acts as a competitive, non-selective antagonist of muscarinic acetylcholine receptors (mAchR), exhibiting relatively equal affinity for all receptor subtypes. Consequently, it inhibits parasympathetic neurotransmission in both the peripheral nervous system (PNS) and the CNS [1,4]. The pharmacokinetic profile of scopolamine is highly dependent on the route of administration. For instance, oral bioavailability is limited, ranging from 10.7% to 48.2%, owing to first-pass metabolism [5], whereas transdermal application offers systemic availability similar to that of intravenous infusion [1]. Therefore, clinically, scopolamine was the first drug formulated in a transdermal therapeutic system, enabling sustained drug release over 72 h. This formulation has demonstrated efficacy in the management of postoperative nausea and vomiting (PONV) [6]. Additionally, scopolamine is used in the prevention and treatment of motion sickness [1,2]. Beyond its role in emesis control, scopolamine can exert a range of physiological effects through parasympathetic inhibition, many of which might have clinical utility. These effects include induction of mydriasis and cycloplegia, reduced salivary, bronchial, and other glandular secretions, or increased heart rate [1]. Moreover, scopolamine-N-butyl bromide has long been used as a spasmolytic agent in abdominal pain or bladder spasms [1].

Within the broader class of tropane alkaloids, scopolamine represents one of several clinically relevant compounds sharing a common bicyclic scaffold. Structurally related agents, including atropine, ipratropium, tiotropium, and tropicamide, have been developed to exploit antimuscarinic activity while optimising tissue selectivity and pharmacokinetic properties. For example, quaternary ammonium derivatives such as ipratropium and tiotropium are restricted to peripheral tissues and are widely used as bronchodilators, whereas compounds such as tropicamide and cyclopentolate are employed in ophthalmology for short-acting mydriasis. Other derivatives, including trospium chloride and N-butylscopolamine, are designed to minimise central nervous system penetration while retaining peripheral spasmolytic activity [4]. These examples highlight the versatility of the tropane scaffold as a pharmacophore and underscore how structural modifications enable dissociation of central and peripheral effects.

Scopolamine administration is often employed as a psychopharmacological model of Alzheimer’s disease (AD), due to its ability to transiently impair cognitive functions by antagonising central mAChR [7]. Interestingly, in this model, scopolamine not only impairs cholinergic neurotransmission but also increases oxidative stress and amyloid β (Aβ) levels, which are additional hallmarks of AD [8]. Emerging evidence suggests that scopolamine may exert rapid and robust antidepressant and anxiolytic effects, likely mediated through its modulation of central cholinergic and glutaminergic neurotransmission [9].

From a biotechnological perspective, recent progress has shifted away from classical total synthesis toward the elucidation of biosynthetic pathways and the development of alternative production strategies. Key advances include the identification of critical enzymatic steps in planta, such as the polyketide synthase-mediated formation of tropinone and the hyoscyamine 6β-hydroxylase conversion of hyoscyamine to scopolamine. Current research focuses on achieving scalable production using plant cell cultures, stable hairy root systems, and heterologous microbial hosts like yeast and *E. coli*. These biologically driven ‘synthesis’ approaches aim to overcome the limitations of traditional agriculture and secure the future pharmaceutical supply [4].

Therefore, this narrative review aims to provide a comprehensive overview of the current knowledge on the pharmacological effects of scopolamine, extending beyond its well-established antiemetic, spasmolytic, and ocular applications. Particular emphasis is placed on its emerging roles in the context of neuroscience and neuropsychiatric disorders.

## 2. Search Strategy

To ensure a comprehensive and methodologically sound review, a literature search was conducted using the PubMed and Google Scholar databases, encompassing publications available up to February 2026. The search strategy included a broad range of study types, including preclinical (experimental), clinical, and review articles. The following keywords were used to identify relevant literature: ‘scopolamine’, ‘mechanism of action’, ‘pharmacodynamics’, ‘antidepressant’, ‘anxiolytic’, ‘neuroprotection’, and ‘neuroplasticity’. Titles and abstracts were screened based on relevance, and additional sources were identified through manual reference list checks to capture potentially overlooked studies. Inclusion criteria were limited to peer-reviewed, full-text articles published in English. Non-peer-reviewed sources, conference abstracts, and non-English publications were excluded to ensure the scientific quality and relevance of the included studies. To minimise selection bias and enhance data reliability, two independent reviewers (J.K. and P.O.) conducted the literature screening and selection process.

## 3. Scopolamine’s Mechanism of Action

### 3.1. Chemical Structure and Stereochemistry of Scopolamine

**Figure 1:**
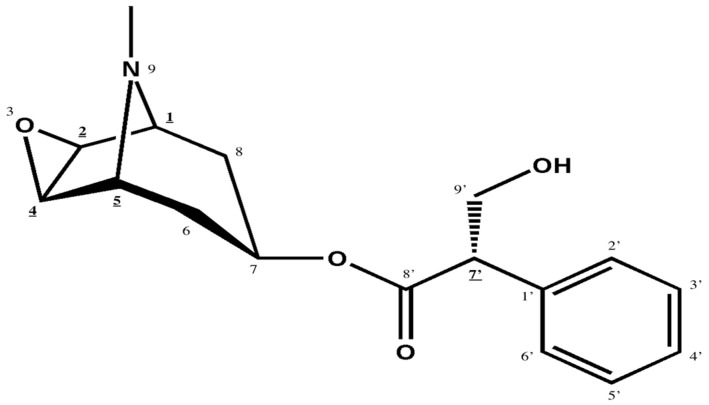
Chemical structure of scopolamine. The chiral centres are underlined.

Structurally, scopolamine is an organic ester of tropic acid with scopine (depicted in Figure 1) [3,4]. The chemical architecture of the scopine moiety resembles the tropine fragment in hyoscyamine, whose racemic mixture is atropine [3,4,10]. A distinguishing feature between scopolamine and hyoscyamine is the presence of an epoxy group oxygen bridge over the carbon-2 and carbon-4 atoms of scopine, which is absent in tropine [10,11]. Five chiral centres are identified in the molecule, and the C-7′ carbon atom, incorporated into the structure of the tropic acid, determines the assignment of D/L configurations (Figure 1). Both levorotatory (−) and dextrorotatory (+) enantiomers of scopolamine exist. Nevertheless, naturally occurring scopolamine consists almost exclusively of L(-)-isomer [10]. This stereoisomer has been shown to exert pharmacological activity in both CNS and PNS [10,12]. Scopolamine has a molecular weight of 303.4 g/mol. Its high lipid solubility facilitates efficient penetration across the blood–brain barrier [3]. Two allosteric (active) sites of the molecule involved in receptor binding have been identified. The first one is the nitrogen group within the aromatic ring, which participates in electrostatic interactions. The second site is the C-7′ carbon atom. The spatial arrangement of those two sites mirrors distances between active sites in acetylcholine molecules; however, the presence of an additional hydroxyl group in hyoscine may further modulate its binding abilities [10].

### 3.2. Pharmacodynamics: mAChR Subtypes and Signalling

The drug is a high-affinity antagonist of the mAChRs, yet it is not particularly selective for any receptor subtype [13]. mAChRs are classified into five subtypes: M1 through M5 (M1R–M5R) [14]. All the above are metabotropic G-protein-coupled receptors (GPCRs) [15]. Thus, they are coupled with specific G proteins (GPs) that play a major role in signal transduction through the cell’s membrane [16]. mAChRs are heptahelical transmembrane proteins, which means they include seven α-helices located within the plasma membrane [15,17]. A 64% to 82% and 82% to 92% similarity in both overall and transmembrane-regions-only sequences, respectively, has been determined in mAChRs [18]. While the transmembrane domain is conserved across all GPCRs, certain phylogenetic groups may possess additional extracellular domains at the N-terminus of the protein. These domains are usually additional binding sites of the native ligands [19]. The crystal structures of all mAChRs subtypes have been determined, with the M5R being the last to be analysed successfully by Vuckovic et al. in 2019 [20]. Each of the mAChR subtypes has at least one allosteric binding site [21]. Moreover, all these subtypes exhibit a high degree of similarity within their active sites [22].

### 3.3. Signal Transduction Downstream of mAChRs

The structure of the heterotrimeric G protein incorporates three subunits: Gα, Gβ and Gγ. Each subunit exists in multiple isoforms. The Gβ and Gγ subunits form a complex that may vary in stability depending on the Gβ subunit type [23]. However, more recent studies suggest that the Gβγ complex does not dissociate under physiological conditions [24]. Within the Gα subunit, a Ras-like domain with intrinsic GTPase activity was identified, along with a nucleotide-binding pocket [24]. The N-terminus of Gα is myristoylated, which promotes binding with the Gβγ complex. Gβγ assembly is attached to the plasma membrane via lipids [25].

M1R, M3R and M5R subtypes are functionally linked to Gq/11 protein subtype, whereas M2R and M4R transduce signal via Gi/0 [26]. Most recent studies indicate that the ligand is attached to the receptor-GP complex, which means that the GP must join the receptor prior to the agonist. Earlier studies supported the model in which agonist binding precedes the association of the GP with the receptor [25,27,28]. This concept was supported by studies focusing on the M3R subtype [27]. It is important to note that GPCRs are not only present in activated or inactivated states but are likely to occur in various intermediate and transitional momentary conformations. This means they are highly dynamic structures changing in response to regulatory proteins, ligands, pH, lipids, ions and GPs [24,29]. Although available studies regarding GPCRs are based mostly on the β2-adrenergic receptor, it was observed with the M2R crystal structure analysis that the mechanism can apply to all GPCRs, including mAChRs. Upon activation, conformation state is being changed, most notably by the movement of the receptor’s transmembrane helix 6 (TM6) together with the structural shift in TM5 and TM7 [28,29].

When bound to the receptor, the GP has the guanosine diphosphate (GDP) attached to its α subunit [23]. The agonist joins the pre-coupled GPCR-GP complex, which provokes the GP to open, so that the GDP is released [27]. The guanosine triphosphate (GTP) replaces GDP in the open GP, which leads to the dissociation of the Gα subunit and the Gβγ complex from each other and from the receptor. In turn, the signal’s transduction is triggered, and eventually ion channels or enzymes are activated or inhibited [23]. Agonist binding enhances GP’s association with the receptor; conversely, the presence of GP also stabilises agonist-receptor interactions [30].

**Figure 2:**
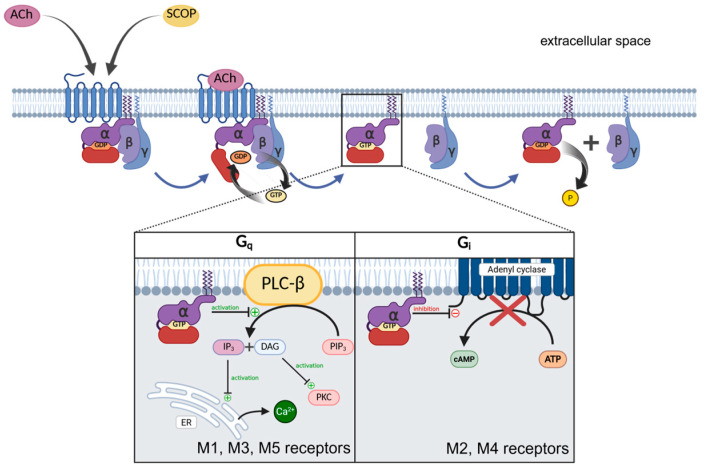
Muscarinic acetylcholine receptor (mAChR) signalling and its antagonism by scopolamine. Acetylcholine (ACh) activates muscarinic G-protein-coupled receptors, triggering exchange of GDP for GTP on the Gα subunit and dissociation of the Gα and Gβγ components. M1, M3, and M5 receptors couple to Gq/11 proteins, activating phospholipase C-β (PLC-β) and generating IP_3_ and DAG, which increase intracellular Ca^2+^ and activate protein kinase C. M2 and M4 receptors couple to Gi/o proteins and inhibit adenylyl cyclase, reducing cAMP production. Scopolamine (SCOP) competitively blocks ACh binding and prevents receptor activation. The figure was created with Biorender.com.

The downstream signalling pathways vary depending on the GP subtype (Figure 2). The Gq/11 protein, coupled with M1R, M3R, and M5R, activates phospholipase Cβ (PLC-β) [31]. This enzyme hydrolyses the phosphatidylinositol 4,5-bisphosphate (PIP2) into inositol triphosphate (IP3) and diacylglycerol (DAG). IP3 activates the calcium channels located in the endoplasmic reticulum, initiating the release of Ca^2+^ ions to the cytosol, while DAG activates the protein kinase C. The Gi/0 protein, associated with M2R and M4R, inhibits the adenyl cyclase, resulting in a decrease in intracellular cyclic adenosine monophosphate (cAMP) concentration [25]. The GTP is hydrolysed as a result of the GTPase activity of the Gα subunit. Thereby, the signal transduction is terminated, allowing the Gα and Gβγ reassociation [23].

### 3.4. The Effects of Scopolamine in CNS and PNS

M1Rs, M4Rs and M5Rs are predominant in the CNS, although other subtypes are also present [20]. M1Rs and M4Rs are involved in the memory-creating process. M1Rs are expressed in neurons of the neocortex and hippocampus, while M4Rs are extensively distributed in the striatum [32,33,34]. M5R subtype is the least studied and is found in dopaminergic neurons of the ventral tegmental area, as well as on the endothelium of cerebral vessels [20]. Central M2Rs are identified in the striatum, hippocampus, and cerebral cortex, where they might exert autoinhibitory effects on acetylcholine-releasing neurons. Reduced acetylcholine levels have been observed in the cerebral cortex and hippocampus of patients with AD. Thus, agents obstructing these receptors may be beneficial in the therapy of this disease by down-regulating the inhibitory action of M2Rs [35,36]. Outside the CNS, M2Rs are key in heart rate regulation, and M3Rs are present within the smooth muscle cells and secretory glands [18,33].

In the CNS, mAChRs are located both pre- and post-synaptically. Different outcomes of mAChR stimulations can be distinguished, including presynaptic inhibition and postsynaptic inhibition or excitation [37]. Presynaptic inhibition is usually determined by M2Rs and M4Rs [16,33]. The postsynaptic response varies and depends on the specific receptor type involved. M2R and M4R usually exhibit an inhibitory effect, while M1R, M3R, and M5R subtypes display excitatory action [16,23].

### 3.5. Mechanistic Basis for Clinical Indications

Scopolamine is well established in PONV and motion sickness management [2,32,38,39]. It is also applied in ophthalmological examination for its pupil dilatating properties, thus inducing mydriasis [11,40]. It is reported that 25 to 30% of patients who had undergone anaesthesia experienced PONV. Several complications of PONV have been determined, such as wound dehiscence and hematoma occurrence. Opioid premedication increases PONV incidence by potentiating neurons of the vestibular nuclei involved in spatial awareness. Gas anaesthetics significantly amplify the probability of PONV event, together with additional factors including female gender, non-smoking, motion sickness history, obesity, surgery time above 3 h, and other administered drugs. Scopolamine effectively alleviates nausea and emesis resulting from the use of opioids during anaesthesia. In some cases, however, the emetogenic effect of drugs such as morphine may persist longer than scopolamine’s therapeutic action. This mismatch may result in delayed PONV development. E0mployment of scopolamine in PONV treatment is limited by adverse effects, such as sedation, blurred vision, xerostomia, hallucinations and memory impairment [38]. Scopolamine is administered via a transdermal patch, applied either the evening before or two hours prior to surgery, delivering 1.0 mg of the drug over 72 h. In motion-related nausea, cholinergic signalling from the vestibular apparatus is transmitted to the vestibular nuclei and then to the nucleus tractus solitarius (NTS) and other components of the brainstem emetic centre. Activation of muscarinic receptors in these regions facilitates the integration of sensory input that triggers nausea and vomiting. Therefore, its antiemetic effect arises from M1R blockage in the vomiting centre as well as from blocking central muscarinic receptors within the vestibular nuclei and associated brainstem pathways. This inhibition reduces the processing of vestibular signals and thereby obstructs the vomiting reflexes [39]. Additionally, the approach of emesis and nausea prevention involves intravenous (IV) administration of 4 to 8 mg of dexamethazone after anaesthesia induction and 4 mg of ondansetron by the end of the surgery [41]. Scopolamine mitigates motion sickness by disrupting sensory integration in the vestibular nuclei. Administered orally (0.3–0.6 mg) or intramuscularly (0.2 mg), it is one of the most effective pharmacological options for motion sickness prevention; however, adverse effects are common in this form of application [42]. For motion sickness prevention, 1 mg transdermal patches of scopolamine are applied on the skin behind the ear 12 h before exposure to motion. Risk factors for motion sickness occurrence comprise female sex, age of 6–12 years old, type of movement, patient’s expectations and genetic and hormonal determinants [43].

## 4. Scopolamine-Induced Cognitive Impairment

### 4.1. Clinical Observations

It has long been clinically established that scopolamine induces anterograde amnesia and sedative effects in healthy volunteers [44]. A study by Patat et al. (1991), involving 12 healthy volunteers, revealed that scopolamine injection (0.6 mg, s.c.), significantly impaired memory performance as assessed by the Buschke Selective Reminding Test, reduced cortical arousal and increased fatigability measured by the Critical Flicker Fusion test and arithmetic performance, and prolonged simple visual reaction time by approximately 80 ms compared with placebo [44]. A study by Nuotto (1983) evaluated the psychomotor, physiological, and cognitive effects of scopolamine administered intravenously at 6 µg/kg and orally at doses up to 0.9 mg in healthy male volunteers under double-blind, placebo-controlled conditions [45]. Intravenous scopolamine produced robust impairments in short-term memory, coordination, reaction time, and subjective alertness, accompanied by bradycardia and reduced systolic blood pressure, whereas oral dosing resulted in comparatively modest effects, primarily affecting flicker fusion performance and memory at higher doses. Notably, concomitant administration of ephedrine mitigated several of the deficits induced by scopolamine, indicating that a proportion of these impairments may be associated with central sedative mechanisms or alterations in noradrenergic signalling, and therefore can be partially alleviated through noradrenergic activation [45]. Similarly, a double-blind, placebo-controlled crossover study conducted by Koller et al. (2003) demonstrated that scopolamine induces broad and dose-dependent impairments in memory functions in young healthy adults (8 females and 4 males; mean age 23.8 ± 2.2 years), with comparatively smaller and less consistent effects on attention [46]. Both doses (0.3 and 0.6 mg, s.c.) significantly disrupted immediate and delayed recall, visual and verbal recognition, and delayed matching performance, while vigilance and simple attentional measures were less uniformly affected [46]. Importantly, scopolamine also produced measurable subjective alterations in the perceived cognitive functioning of healthy individuals across the trials [44,45]. Also, a resting-state magnetoencephalographic connectivity study conducted by Bajo et al. (2015) examined whether scopolamine administration (0.3 mg, i.v.) in healthy elderly individuals induces network-level alterations comparable to those observed in AD compared to placebo [7]. The findings demonstrated increased phase synchronisation in the delta band, consistent with pathological cortical slowing, accompanied by reduced functional connectivity in the alpha, beta, and gamma bands, which are critical for large-scale neural integration and higher-order cognitive processing [7]. The most recent systematic review and meta-analysis conducted by Miravalles et al. (2025), encompassing 46 studies in healthy adults, evaluated the effects of scopolamine administered via various routes on cognitive tasks related to memory and attention [47]. The analysis has shown that scopolamine consistently impairs both memory and attentional performance. Higher doses were reliably associated with marked deterioration in memory and attentional outcomes, whereas lower doses also induced significant impairments in specific domains, particularly immediate and delayed free recall as well as choice reaction time. In contrast, certain measures such as forward digit span remained unaffected, and performance on tasks including backward digit span, recognition memory, and simple reaction time showed dose-dependent vulnerability, with deficits emerging selectively at either higher or lower dose ranges, indicating task-specific sensitivity to scopolamine exposure. Finally, more pronounced cognitive deficits were observed following parenteral administration compared with non-injectable routes, likely reflecting the pharmacokinetic profile of scopolamine and the reduced systemic bioavailability associated with alternative routes of administration [47]. Altogether, these findings indicate that scopolamine administration, particularly via injectable routes, represents a well-validated and experimentally robust model for inducing transient cognitive dysfunction that parallels key features of dementia [36,47].

### 4.2. Proposed Mechanistic Link

Scopolamine induces a rapid and reversible ‘cholinergic lesion’ that has long been used to test the cholinergic hypothesis linking reduced cortical and hippocampal cholinergic tone to memory failure in AD [36,48]. This hypothesis subsequently pursued the development of acetylcholinesterase (AChE) inhibitors as therapeutic strategies for the management of memory deficits and cognitive dysfunction in patients with mild to moderate AD [49]. However, the effect of scopolamine is not restricted to cholinergic dysfunction. Its effects involve mitochondrial dysfunction, oxidative stress, neuroinflammation, and suppression of neurotrophic factors, but also induce Aβ and tau pathology as well as apoptosis of neural cells, leading to brain atrophy [50]. According to Safar et al. (2016), daily administration of scopolamine (2 mg/kg/day, i.p.) for 6 weeks in male Wistar rats significantly increased Aβ protein levels and amyloid precursor protein (APP) mRNA expression compared to control rats receiving only vehicle (saline, p.o.) [51]. On the other hand, it decreased mRNA expression of neprilysin, which is a zinc-dependent metalloprotease involved in the degradation of Aβ, in comparison to the control. Scopolamine administration significantly increased the levels of pro-inflammatory cytokines, IL-1β and TNF-α, whereas it significantly decreased the level of anti-inflammatory IL-10. Scopolamine has also significantly decreased levels of vascular endothelial growth factor (VEGF), nerve growth factor (NGF), brain-derived neurotrophic factor (BDNF), and downregulated the neuroprotective nuclear factor erythroid 2-related factor (Nrf2) [51]. A study by Hsieh et al. (2003) revealed that intracerebroventricular administration of scopolamine (10 mg/kg) to male Sprague-Dawley rats significantly alters the expression of AD-related genes, characterised by decreased tau expression and concomitant upregulation of amyloid-associated genes, suggesting activation of pro-amyloidogenic molecular pathways [52]. Scopolamine administration to male Kunming mice (at 2 mg/kg, i.p.) significantly enhances oxidative stress in the hippocampus, as demonstrated by Hou et al. (2014) whose study has shown a pronounced decrease in superoxide dismutase activity and glutathione concentration, together with a significant elevation in malondialdehyde levels in hippocampus relative to vehicle-treated controls (receiving corresponding volumes of saline, i.p.), indicating impaired antioxidant defence and increased lipid peroxidation [53]. Chronic scopolamine administration (1 mg/kg, i.p., daily for 21 days) in old (aged 18–24 months) female Wistar albino rats enhances the oxidative stress and intracellular Ca^2+^ accumulation in hippocampal neurons, accompanied by mitochondrial membrane depolarization and increased apoptosis, changes associated with transient receptor potential melastatin 2 (TRPM2) and transient receptor potential vanilloid 1 (TRPV1) channel activation [54]. Therefore, scopolamine induces a pro-apoptotic molecular profile in the hippocampus, characterised by upregulation of the pro-apoptotic protein Bax and concomitant downregulation of the anti-apoptotic protein Bcl-2, thereby increasing the Bax/Bcl-2 ratio and indicating activation of intrinsic mitochondrial apoptotic pathways [53]. Daily administration of scopolamine (at 2 mg/kg/day, i.p., for 6 weeks) in male Wistar rats produced marked alterations in hippocampal neurotransmission that extended beyond the cholinergic system. As scopolamine significantly reduced acetylcholine, dopamine, and GABA hippocampal levels, while concomitantly increasing acetylcholinesterase activity and glutamate levels compared with the control group receiving only vehicle (saline, p.o.) [51]. Previous experimental studies showed that the amnestic effects of scopolamine are not exclusively related to cholinergic mechanisms. In addition to impairing memory retention, scopolamine can disrupt both the acquisition of information and memory processing. Although these deficits can be attenuated by cholinergic agonists and cholinomimetic agents, similar protective effects have also been observed with compounds acting on other neurotransmitter systems, including noradrenergic, serotonergic, glycinergic, and GABAergic pathways, suggesting that scopolamine-induced behavioural impairments involve broader neurochemical mechanisms rather than a purely cholinergic deficit [55].

## 5. Antidepressant Effects of Scopolamine and Potential Impact on Neuroplasticity

### 5.1. Clinical Observations

Initial research on the use of scopolamine in depression dates back to the late 1980s and early 1990s [56,57]. In 1988 Newhouse an co-workers conducted a double-blind, multidose study, involving nine elderly patients with major depression undergoing five separate test sessions, receiving on each occasion an injection of scopolamine (0.1, 0.25, or 0.5 mg, i.v.) or placebo, along with an oral dose of lorazepam (1 mg, p.o.) or placebo to assess sensitivity to muscarinic cholinergic blockade. Significant cognitive and behavioural impairments, including deficits in attention, new learning, semantic retrieval, and increased activation, were observed only at the highest dose of scopolamine, while mood and depressive symptoms remained unaffected [57]. Later on, Gillin et al. (1991) conducted the first well-controlled study, which showed that scopolamine (three doses of 0.4 mg, i.m.) exerts a little though significant antidepressant effect, with no withdrawal rebound effect observed [56]. Furey and Drevets (2006) evaluated the antidepressant efficacy of scopolamine (three doses of 4.0 μg/kg each via a 15-min i.v. infusion) using double-blind, randomised, placebo-controlled clinical trial designs in patients with major depressive disorder and bipolar depression [58]. They revealed that scopolamine produced rapid and statistically significant reductions in depressive and anxiety symptom severity, with therapeutic effects emerging within several days of treatment. Nonetheless, clinical improvements were sustained beyond active drug administration, indicating effects that extend beyond acute pharmacologic action [58]. Furthermore, another double-blind, randomized, placebo-controlled, crossover clinical trial by Furey and co-workers, involving 52 individuals suffering from recurrent major depressive or bipolar disorder, revealed that although scopolamine (seven doses of 4.0 μg/kg each via a 15-min i.v. infusion) induces rapid and significant reduction in depressive symptoms in both males and females, the antidepressant and antianxiety response is significantly greater in female participants compared with males [59]. Jaffe et al. (2013) conducted a systematic review that included seven studies investigating the antidepressant effects of scopolamine [60]. The vast majority of the included studies reported that scopolamine produced rapid and clinically significant improvements in both major depressive disorder and bipolar depression [60]. On the other hand, in a randomised, placebo-controlled, crossover trial conducted by Park et al. (2019), scopolamine (three doses of 4.0 μg/kg, i.v.) induced no significant antidepressant or anxiolytic effects in medication-free major depressive disorder individuals in comparison with placebo [61]. BDNF plasma concentrations were not significantly affected by scopolamine administration, and there was no correlation between BDNF changes and changes in the Montgomery–Asberg Depression Rating Scale [61]. Similarly, in a randomized, active placebo-controlled trial, a single dose of scopolamine hydrobromide (4 to 6 μg/kg, i.v.) produced no significant antidepressant effects compared with glycopyrronium bromide in subjects with major depressive disorder, with mixed-effects and Bayesian analyses supporting the absence of efficacy despite reductions in depressive symptoms in both groups of participants as measured by Montgomery–Åsberg Depression Rating Scale [62]. Finally, in a randomised, placebo-controlled trial exclusively involving individuals with bipolar disorder experiencing depressive episodes, scopolamine (three doses of 4.0 μg/kg, i.v.) also failed to demonstrate significant antidepressant efficacy compared with saline placebo, despite overall symptom improvement observed in both groups of patients [63]. Overall, findings regarding the antidepressant efficacy of scopolamine remain inconsistent, with early studies suggesting rapid symptom improvement, whereas more recent rigorously controlled trials employing active or saline placebos have yielded largely null or inconclusive results.

### 5.2. Proposed Mechanistic Link

The hypothesis implicating cholinergic dysfunction in the pathophysiology of depression was first articulated by Janowsky et al. in 1972, who proposed that depressive states may reflect central cholinergic predominance, whereas manic symptoms may arise from relative adrenergic and serotonergic dominance [64]. Evidence suggests that exposure to acetylcholinesterase inhibitors, including organophosphate pesticides, is associated with psychiatric symptoms, particularly depression. Similarly, pharmacological enhancement of cholinergic transmission via acetylcholinesterase inhibitors, such as donepezil, has been associated with increased depressive relapse and greater severity of affective symptoms. Administration of acetylcholine precursors, including choline, deanol, and lecithin, has been reported to precipitate depressive symptoms and, in some cases, hypomanic episodes, particularly among individuals with a prior history of affective disorders, further implicating heightened cholinergic tone in mood destabilisation. Converging neuroimaging evidence further suggests elevated central acetylcholine levels in unipolar and bipolar depression, collectively supporting a model in which cholinergic hyperactivity contributes to depressive symptom expression and mood destabilisation [65].

However, the antidepressant effects of scopolamine are not solely attributable to its antimuscarinic properties or its direct modulation of cholinergic neurotransmission. Preclinical evidence indicates that the antidepressant effects of scopolamine are associated with enhanced glutamatergic transmission and increased synaptogenesis, parallel to N-methyl-D-aspartate receptor (NMDAR) antagonists, such as ketamine [66]. According to Voleti et al. (2013), scopolamine administration (at 25 µg/kg, i.p., which corresponds with the low dose of scopolamine used in the trials involving patients with major depressive disorder 4 µg/kg) to male Sprague–Dawley rats rapidly activates mammalian target of rapamycin complex 1 (mTORC1) signalling and therefore increases dendritic spine density and synaptic function in layer V pyramidal neurons of the prefrontal cortex (PFC), which are canonical markers of structural and functional synaptic plasticity [66]. Additionally, scopolamine’s antidepressant-like behavioural effects can be blocked by mTORC1 or α-amino-3-hydroxy-5-methyl-4-isoxazolepropionic acid (AMPA) receptor inhibition [66]. Interestingly, according to Rami et al. (1997), scopolamine did not protect hippocampal CA1 neurons from ischemic injury in vivo in male Wistar rats subjected to transient forebrain ischemia, despite a clear dorsal to ventral gradient of vulnerability in the hippocampus, with the dorsal region showing markedly greater neuronal loss [67]. In this study, scopolamine (0.1, 1 and 3 mg/kg, i.v.) was administered 15 min prior to ischemia. However, their study found that while scopolamine failed to reduce neuronal necrosis after global ischemia in rats or focal ischemia in male NMRI mice subjected to permanent middle cerebral artery (MCA) occlusion, it significantly improved neuronal survival in primary hippocampal cultures (derived from neonatal Fischer 344 rats) exposed to glutamate, suggesting a context-dependent interaction between muscarinic and NMDA receptor mediated mechanisms [67].

Navarria et al. (2015) demonstrated that the medial PFC constitutes a critical locus for scopolamine’s antidepressant actions, showing that microinfusion into prelimbic or infralimbic regions induces antidepressant-like responses and that selective M1 muscarinic receptor antagonism stimulates mTORC1 signalling and reverses stress-induced anhedonia in male Sprague–Dawley rats [68]. According to Wohleb et al. (2016), in transgenic C57BL/6 mice, the rapid antidepressant-like effects of scopolamine (3 injections of 25 μg/kg, i.p., every 48 h) are mediated specifically by M1 muscarinic acetylcholine receptors located on GABAergic interneurons in the medial PFC. Importantly, knockdown of M1 receptors specifically in somatostatin-positive interneurons abolished the behavioural response to scopolamine [69].

In parallel, Liu et al. (2021) reported that M2 muscarinic acetylcholine receptors in the medial PFC also contribute to scopolamine’s antidepressant-like effects (25 μg/kg, i.p.), as M2 antagonism mimicked scopolamine’s behavioural actions and increased BDNF expression and mTORC1 signalling, whereas M2 knockdown attenuated these effects in SPF Sprague Dawley (SD) male rats [70]. Ghosal et al. (2018) showed that activity-dependent BDNF release within the medial PFC is required for scopolamine’s rapid antidepressant actions, as behavioural effects were abolished in BDNF Val/Met knock-in mice and by intra-prefrontal infusion of a BDNF-neutralising antibody, implicating tropomyosin receptor kinase B (TrkB) and downstream signalling pathways involvement [71]. According to Petryshen et al. (2016), co-administration of subeffective doses of ketamine and scopolamine produced a robust antidepressant-like response in the forced swim test, whereas each drug alone at the same doses was ineffective, indicating a synergistic interaction in male C57BL/6N mice [72]. This finding supports the hypothesis that NMDA receptor antagonism and muscarinic receptor blockade may converge on shared downstream pathways, as both agents enhance glutamatergic transmission in the PFC, leading to AMPA receptor activation. This process promotes activity-dependent BDNF release and TrkB receptor engagement, which subsequently stimulates mTORC1 signalling pathways involved in synaptic plasticity. These processes are thought to underlie the rapid antidepressant effects. In the context of potential coadministration, this mechanism may allow lower individual doses and improved tolerability [73].

## 6. Therapeutic Implications and Future Research Perspectives

Scopolamine presents a clinically provocative paradox: acute cognitive disruption coexisting with potential rapid antidepressant and neuroplastic effects. Therapeutically, this suggests that muscarinic modulation should not be conceptualised as globally deleterious or beneficial, but rather as dose-dependent, circuit-specific, and receptor subtype-selective. Future drug development should prioritise selective targeting of M1 and M2 receptors, particularly within medial prefrontal networks, to preserve or enhance synaptic plasticity while minimising hippocampal-dependent memory impairment. Precise pharmacology approaches, including biased ligands or regionally restricted delivery strategies, may help dissociate antidepressant relevant glutamatergic and mTORC1 signalling cascades from widespread cholinergic blockade. Clinically, resolving the inconsistent antidepressant findings requires adequately powered trials with rigorous blinding procedures and active placebos that mimic peripheral anticholinergic effects. An analysis of ClinicalTrials.gov records indicates that, although several studies investigating scopolamine have already been completed, a subset of trials remains active. Among the identified studies, 13 are currently recruiting participants, 2 are active but not recruiting, and 1 is enrolling by invitation only. This distribution confirms that, despite a number of finished trials, clinical research on scopolamine is ongoing, with multiple studies still open to participant inclusion. Notably, several trials continue to explore established indications, such as motion sickness and procedural analgesia, including studies like NCT05886660, which evaluates intranasal scopolamine combined with sensory augmentation to mitigate G-transition-induced motion sickness and enhance sensorimotor performance, and NCT07383571, comparing oral hyoscine with topical anaesthetics for pain reduction during hysterosalpingography, reflecting its peripheral antimuscarinic effects on smooth muscle. In parallel, emerging neuropsychiatric applications are also being investigated, as exemplified by NCT05594017, a randomised crossover study in patients undergoing intracranial electroencephalographic monitoring for resistant epilepsy. In this protocol, intravenous scopolamine is administered in a controlled setting to examine its effects on episodic and spatial memory performance, alongside high-resolution electrophysiological recordings, enabling direct assessment of its impact on hippocampal oscillatory dynamics and memory-related neural circuitry. Stratification by sex, depression subtype, bipolarity, baseline cognitive status, and potentially cholinergic tone could identify responsive subgroups. Integration of translational biomarkers, such as electrophysiological connectivity measures, neurotrophin profiles, and molecular indicators of mTORC1 engagement, would allow mechanistic validation rather than reliance on symptom scales alone. Combination strategies also warrant systematic exploration. Subthreshold co-administration with glutamatergic modulators, such as NMDA receptor antagonists, or noradrenergic agents, may amplify rapid synaptic plasticity while permitting lower doses that reduce cognitive liability. At the same time, careful safety frameworks are essential, particularly in older adults or cognitively vulnerable populations, to mitigate risks of confusion, autonomic instability, and potential pro-inflammatory or oxidative consequences with repeated exposure. From a translational standpoint, scopolamine should be viewed less as a standalone therapeutic and more as a mechanistic template, informing the development of next-generation agents that harness rapid neuroplastic pathways without incurring broad cognitive cost. Finally, there is limited evidence indicating that sustained administration of scopolamine during the latent phase following status epilepticus may decrease the frequency of spontaneous recurrent seizures, mitigate cognitive and behavioural deficits, and reduce aberrant mossy fibre sprouting, as demonstrated by Meller et al. (2021) in a rat model of mesial temporal lobe epilepsy [74]. The authors suggest that scopolamine may exert disease-modifying or antiepileptogenic effects, potentially through modulation of cholinergic signalling pathways implicated in the mechanisms underlying epileptogenesis after brain injury [74].

## 7. Conclusions

Scopolamine remains a powerful experimental model linking cholinergic modulation to memory dysfunction and rapid synaptic plasticity. Its dual profile, cognitive impairment alongside potential antidepressant related neuroplastic effects, underscores the need for receptor-specific, circuit-informed, and biomarker-guided approaches to translate its mechanistic insights into safer and more precise neuropsychiatric therapies.

## Data Availability

All data supporting the findings of this study are available within the paper and can be accessed by DOI from references.

## Abbreviations

The following abbreviations are used in this manuscript:

Aβ	amyloid β
ACh	acetylcholine
AChE	acetylcholinesterase
AD	Alzheimer’s Disease
AMPA	α-amino-3-hydroxy-5-methyl-4-isoxazolepropionic acid
APP	amyloid precursor protein
BDNF	brain-derived neurotrophic factor
cAMP	cyclic adenosine monophosphate
CNS	central nervous system
DAG	diacylglycerol
GABA	gamma-aminobutyric acid
GDP	guanosine diphosphate
GP	G protein
GPCR	G-protein-coupled receptor
GTP	guanosine triphosphate
IP3	inositol triphosphate
M1R–M5R	muscarinic type 1–5 receptors
mAChR	muscarinic acetylcholine receptor
mTORC1	mammalian target of rapamycin complex 1
NGF	nerve growth factor
NMDAR	N-methyl-D-aspartate receptor
Nrf2	nuclear factor erythroid 2-related factor
NTS	nucleus tractus solitarius
PDNV	post discharge nausea and vomiting
PFC	prefrontal cortex
PIP2	phosphatidylinositol 4,5-bisphosphate
PLC-β	phospholipase Cβ
PNS	peripheral nervous system
PONV	postoperative nausea and vomiting
ROS	reactive oxygen species
SCOP	scopolamine
TM6	transmembrane helix 6
TrkB	tropomyosin receptor kinase B
TRPM2	transient receptor potential melastatin 2
TRPV1	transient receptor potential vanilloid 1
VEGF	vascular endothelial growth factor
